# Paeonolum protects against MPP^+^-induced neurotoxicity in zebrafish and PC12 cells

**DOI:** 10.1186/s12906-015-0661-0

**Published:** 2015-04-29

**Authors:** Xi-Lin Lu, Yue-Hao Lin, Qi Wu, Feng-Juan Su, Cheng-Hui Ye, Lei Shi, Bai-Xuan He, Fei-Wen Huang, Zhong Pei, Xiao-Li Yao

**Affiliations:** Department of Neurology, Guangdong Key Laboratory for Diagnosis and Treatment of Major Neurological Diseases, First Affiliated Hospital, Sun Yat-sen University, Guangzhou, 510080 China; National Key Clinical Department, Guangdong Key Laboratory for Diagnosis and Treatment of Major Neurological Diseases, First Affiliated Hospital, Sun Yat-sen University, Guangzhou, 510080 China; Key Discipline of Neurology, Guangdong Key Laboratory for Diagnosis and Treatment of Major Neurological Diseases, First Affiliated Hospital, Sun Yat-sen University, Guangzhou, 510080 China; Department of Clinical Laboratory, Cancer Center of Sun Yat-sen University, Guangzhou, 510060 China; Department of Geriatrics, First Affiliated Hospital, Sun Yat-sen University, Guangzhou, 510080 China; Neurosurgery Intensive Care Unit, Department of Critical Care Medicine, First Affiliated Hospital, Sun Yat-sen University, Guangzhou, 510080 China

**Keywords:** Paeonolum, MPP^+^, Mitochondrial, Zebrafish, Total GSH, ROS

## Abstract

**Background:**

Parkinson’s disease (PD) is the second most common neurodegenerative disease, affecting 2% of the population aged over 65 years old. Mitochondrial defects and oxidative stress actively participate in degeneration of dopaminergic (DA) neurons in PD. Paeonolum, a main component isolated from Moutan cortex, has potent antioxidant ability. Here, we have examined the effects of paeonolum against MPP^+^-induced neurotoxicity in zebrafish and PC12 cells.

**Methods:**

The overall viability and neurodegeneration of DA neurons was assessed in ETvmat2:green fluorescent protein (GFP) transgenic zebrafish, in which most monoaminergic neurons are labeled by GFP. Damage to PC12 cells was measured using a cell viability assay and assessment of nuclear morphology. Intracellular reactive oxygen species (ROS) and the level of total GSH were assessed. The mitochondrial cell death pathway including mitochondrial membrane potential, cytochrome C release and caspase-3 activity were also examined in PC12 cells.

**Results:**

Paeonolum protected against MPP^+^-induced DA neurodegeneration and locomotor dysfunction in zebrafish in a concentration-dependent manner. Similar neuroprotection was replicated in the PC12 cellular model of MPP^+^ toxicity. Paeonolum attenuated MPP^+^-induced intracellular ROS accumulation and restored the level of total GSH in PC12 cells. Furthermore, paeonolum significantly inhibited the mitochondrial cell death pathway induced by MPP^+^.

**Conclusions:**

Collectively, the present study demonstrates that paeonolum protects zebrafish and PC12 cells against MPP^+^-induced neurotoxicity.

## Background

Parkinson’s disease (PD) is the second most prevalent neurodegenerative disease, affecting 2% of the population aged over 65 years. Clinically, PD patients mainly present with impairments in movement including: rigidity, resting tremor and postural instability [1,2]. Current treatments only reduce symptoms, but do not stop the progression of PD. Therefore, there is an urgent need to find more appropriate treatments that can prevent the PD progression.

Although the pathogenesis of PD remains largely unknown, both oxidative stress and mitochondrial dysfunction play pivotal roles in the pathogenesis of PD. For example, a defect in respiratory chain complex I activity in mitochondria has been observed in dopaminergic (DA) neurons in PD patients [3-7]. It is generally believed that both genetic susceptibility and environmental factors contribute to the etiology of PD. However, the majority of PD cases are sporadic, indicating a critical role for environmental factors in the pathogenesis of PD. 1-methyl-4-phenyl-1,2,3,6-tetrahydropyridine (MPTP) is an environmental toxin and can selectively damage DA neurons in the substantia nigra (SN) which in turn leads to clinical symptoms similar to those of PD in humans [8,9]. Thus, MPTP and its metabolite MPP^+^ have been widely used to model PD in mammals [1,2,9,10].

Recently, model organisms such as zebrafish, are becoming powerful tools to study human diseases. Many of the basic physiological processes, metabolic signaling pathways and neurotransmitters in humans are conserved in such models [11,12]. Because model organisms are easy to grow and cost-effective to maintain, their use as a high-throughput strategy to identify new drug targets for human diseases is increasing. Recently, a zebrafish model of MPP^+^ toxicity has been established that can recapitulate many key features of PD including the specific degeneration of DA neurons and impairment of movement [13]. This model has been increasingly used as an *in vivo* tool for screening neuroprotective agents for PD.

MPP^+^-mediated neurotoxicity is believed to be mediated by inhibition of complex I activity and excessive formation of reactive oxygen species (ROS) [1,2,14]. Many antioxidants and agents that can protect the mitochondria have been reported to reduce MPTP-induced neurotoxicity and some have shown promising results in clinical trials with PD patients [2,15].

In recent years, natural antioxidants have become attractive targets for drug development in neurodegenerative diseases because of their demonstrated neuroprotective effects in animal models and low levels of toxicity. Paeonolum is a main component isolated from Moutan cortex. Paeonolum has been used as an anti-inflammatory drug for thousands of years in China. In addition to anti-inflammatory actions, paeonolum has many other pharmacological properties including antiproliferative and antioxidative actions. Most importantly, paeonolum can easily cross the blood–brain barrier (BBB) which makes it an attractive compound for central nervous system (CNS) drug development [16]. The present study was conducted to investigate the neuroprotective potential of paeonolum against MPP^+^-induced neurotoxicity in zebrafish and PC12 cells.

## Methods

### The isolation of paeonolum

The root bark of *Paeonia suffruticosa* belongs to Ranunculaceae. Paeonolum was isolated and purified by the State Key Laboratory of Quality Research in Chinese Medicine, Institute of Chinese Medical Sciences, University of Macau, China, according to a published method [17]. The purity of the compound was >98%. Paeonolum was dissolved in dimethyl sulfoxide (DMSO) and stored at −20°C until use. The solution form of paeonolum was then diluted by PBS to the concentration needed. All reagents were purchased from Sigma (Sigma, Shanghai, China) unless otherwise stated.

### Zebrafish MPP^+^ model and paeonolum treatment

The ETvmat2:green fluorescent protein (GFP) transgenic zebrafish was identified from a large-scale enhancer trap screen using a Tol2 vector containing a 249-bp zebrafish gata2 minimal promoter linked to a GFP reporter gene [18,19]. The fish line was maintained in a recirculating aquaculture system at 28.5°C. The MPP^+^ (Sigma-Aldrich) was dissolved in distilled water to 40 mM and was used at a final concentration of 100–1000 μM for the treatment. Embryos were obtained from the ETvmat2:GFP transgenic fish. At 22–24 hours post-fertilization (hpf), GFP-positive embryos were collected and dechorionated. Ten embryos were then transferred into a six-well plate containing 4 ml Holtfreter’s buffer with 0.003% phenylthiourea (Sigma-Aldrich) and MPP^+^. The MPP^+^-containing buffer was changed once after 2 days and at 5 days post-fertilization (dpf), the larval zebrafish were examined under a fluorescence microscope.

### Testing of the locomotor activity

An automated computer tracking system was used to determine the locomotor activity of zebrafish larvae (Viewpoint Life Sciences Inc.). At 4 dpf, larval zebrafish were transferred to 96-well microplates and acclimated at 28°C overnight for 24 h. The 96-well microplates were then put into the zebrafish tracking box (Viewpoint Life Sciences Inc.) and activity of the zebrafish monitored using an automated video-tracking system (Viewpoint video-tracking system and software). The speed corresponding to the total length traveled by the larvae was divided by time and then analyzed (Viewpoint software). The results represent the mean swim speed of 10 larvae and are expressed in mm/s.

### Morphology assay

Briefly, zebrafish (5 dpf) with or without compounds were anaesthetized by treatment with 0.4% tricaine and mounted onto glass slide. Overall morphology of the zebrafish was visualized at 4× magnification under an OLYMPUS IX71 inverted microscope.

### Cell culture and cell viability assay

PC12 (rat pheochromocytoma) cells were cultured in RPMI-1640 (Invitrogen, USA) supplemented with 10% heat-inactivated horse serum (Invitrogen, USA) and 5% heat-inactivated fetal calf serum (Invitrogen, USA) in an humidified incubator (Thermo electron corporation, USA) with 5% CO_2_ at 37°C. PC12 Cells were differentiated on collagen-coated plates by treating with 100 ng/ml nerve growth factor for 9 days and then washed with RPMI-1640 medium containing 1% fetal bovine serum, 24 h before experiments. Cellular viability was assessed using the MTT kit (MTT Cell Growth Kit, Chemicon, USA) according to the manufacturers instructions. Briefly, PC12 cells were diluted to 1 × 10^5^ cells/mL and plated at 100 μL per well in a 96-well microplate, then treated with various concentrations of paeonolum and/or MPP^+^ (250 μM) for 24 h. 10 μL of the MTT labeling reagent (0.5 mg/mL) was added into each well at 24 h after MPP^+^ treatment and cells were further incubated with MTT in an humidified incubator at 37°C with 5% CO_2_ and 95% air (v/v) at 90% humidity for 4 h to allow formation of purple formazan crystal. Four hours later, 100 μL of solubilization reagent was added to each well. Finally, the absorbance of the solubilized purple formazan crystals was measured using a microplate spectrophotometer at an absorbance wavelength of 570 nm. In order to examine MPP^+^-induced nuclear morphology, PC12 cells were stained with the fluorescent dye, DAPI (2.5 μg/ml, Sigma, USA). PC12 cells (1 × 10^5^ cells/ml) were treated with MPP^+^ alone or in the presence of paeonolum for 24 h at 37°C and changes in nuclear morphology of the cells were assessed by microscopic observation. Briefly, PC12 cells were fixed in 4% polylysine on ice for 15 min and were then washed twice with PBS. The cells were then incubated with DAPI in PBS for 20 min at room temperature. Nuclear morphology of cells stained with DAPI was visualized under a fluorescence microscope (Olympus DP70, Japan) and quantification was performed by a person who was blinded to the experimental treatment. Condensed, shrunken, fragmented or irregular nuclei were considered signs of cell death. To quantitatively analyze changes in nuclear morphology, the percentage of cells with abnormal nuclei was calculated. All cells from 10 random microscopic fields at 40× magnification were counted and more than 500 cells in total were observed for each treatment group. The ratio of dead cells to total number of cells was then compared among the different treatment groups. The results were obtained from four independent experiments performed in triplicate.

### Measurement of intracellular reactive oxygen species

Intracellular ROS were measured by using the fluorescent probe 2,7-dichlorofluorescein diacetate (DCF-DA). The dye DCF-DA is oxidized to fluorescent 2,7-dichlorofluorescin (DCF) by intracellular hydrogen peroxide or low-molecular-weight peroxides. PC12 cells (1 × 10^5^ cells/ml) were seeded in 96-well plates and incubated with MPP^+^ alone or in the presence of paeonolum for 24 h. Cells were incubated with 10 μM DCF-DA at 37°C for 30 min and then washed twice with PBS. Quantitative measurements of fluorescence readings were determined at 485/538 nm (excitation/emission) using a SpectraMax GEMINI EM fluorescent plate reader (Molecular Devices, USA) from cells in 96-well plates. The results were obtained from three independent experiments performed in triplicate.

### Measurement of mitochondrial membrane potential

Mitochondrial membrane potential was measured using the fluorescence dye rhodamine 123 (Invitrogen). Cells were exposed to 10 μM rhodamine 123 for 25 min and then washed twice with HBSS before the fluorescence was determined with a SpectraMax GEMINI EM fluorescent plate reader (Molecular Devices, USA) at 511/534 nm. The results were obtained from three independent experiments performed in triplicate.

### Measurement of total glutathione level

The level of total glutathione (reduced form GSH + oxidized form GSSG) was determined using glutathione reductase. PC12 cells were treated with MPP^+^ alone or in the presence of paeonolum for 24 h at 37°C, centrifuged at 412 × g for 10 min in a microplate centrifuge, the media removed and cells were washed twice with PBS. Cells were dissolved with 2% 5-sulfosalicylic acid (100 μl) and incubated in 100 μl of the reaction mixture containing 22 mM sodium EDTA, 600 μM NADPH, 12 mM DTNB and 105 mM NaH_2_PO_4_, pH 7.5 at 37°C. Glutathione reductase (20 μl of 100 U/ml) was added, and the mixture incubated for an additional 10 min. Absorbance was measured at 412 nm using a microplate spectrophotometer. A standard curve was obtained from absorbance of the diluted commercial GSH that was incubated in the mixture as in samples. The results were obtained from three independent experiments performed in triplicate.

### Measurement of caspase-3 activity

Caspase-3 activity was determined by using the caspase-3 activity assay kit (Life Science, USA). Briefly, after treatment, cells were harvested and washed with cold PBS. Each cell sample was resuspended in cell lysis buffer (100 mM HEPES, pH 7.4, 10% sucrose, 0.1% CHAPS, 1 mM EDTA, 10 mM dithiothreitol, 1 mM PMSF, 10 mg/ml pepstain, and 10 mg/ml leupeptin) and left on ice for 40 min. The lysed cells were then centrifuged to pellet the cellular debris. The supernatant containing 50 mg protein was incubated with 5 μM fluorogenic substrate peptide Z-DEVD-ER110 in 100 μl of lysis buffer. The intensity of fluorescence of Z-DEVD-ER110 substrate was measured in a fluorescence microplate reader using excitation at 496 nm and emission detection at 520 nm.

### Immunoblot analysis for cytochrome c

After treatment, cells were collected and washed with PBS, then resuspended in ice-cold homogenizing buffer (250 mM sucrose, 1 mM dithiothreitol, 1 mM phenylmethylsulfonylfluoride, 1 mg/mL aprotinin and 1 mg/mL leupeptin). Cells were homogenized with a glass Dounce homogenizer. The homogenate was centrifuged at 1000 rpm for 10 min. Then the resulting supernatant was centrifuged at 10,000 rpm (4°C, first for 10 min, and then for 1 h), to produce cytosol. 50 mg of each protein sample was subjected to SDS-polyacrylamide gel electrophoresis on a 12% gel. Then the protein was transferred onto a nitrocellulose membrane and immunoblotted with cytochrome c antibody as previously described [20]. Finally samples were incubated with goat anti-mouse IgG-horseradish peroxidase. The densitometric analysis of protein bands was performed using the image J analysis system.

### Statistics

Data are presented as mean ± SEM. One-way ANOVA was performed followed by a Tukey post-hoc test to compare the significance between the individual groups. P-values less than 0.05 were considered significant.

All experimental procedures were approved by the Institutional Animal Ethical Committee of Sun Yat-sen University and were conducted according to the Guide for the Care and Use of Laboratory Animal of the National Institute of Health (Publication No. 80–23, revised 1996).

## Results

### Paeonolum attenuated MPP^+^-induced neurotoxicity in zebrafish

**Figure 1:**
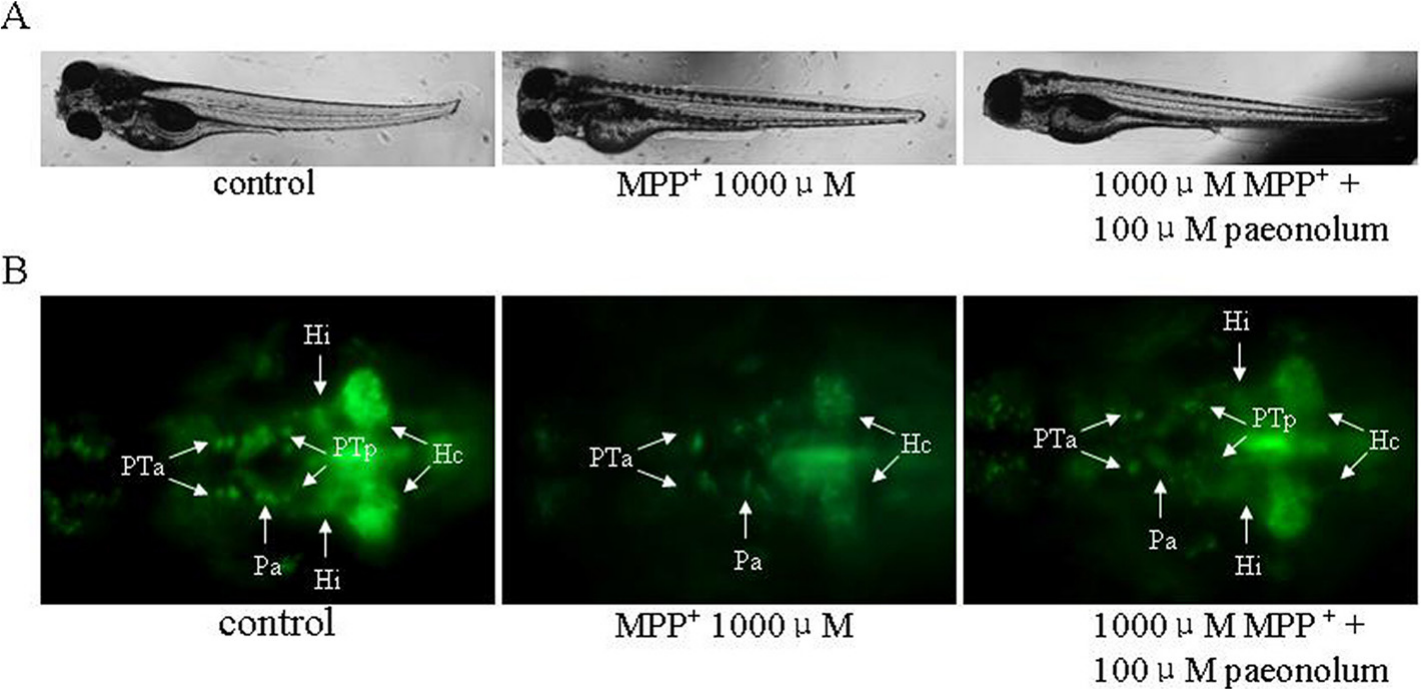
Paeonolum attenuated the MPP^+^-induced neurotoxicity in zebrafish. **(A)** Representative brightfield images of zebrafish larvae at 5 dpf. Treatment with MPP^+^ or paeonolum for up to 5 dpf did not adversely affect the normal development of zebrafish larvae. **(B)** Paeonolum attenuated MPP^+^-induced DA neurotoxicity in zebrafish larvae.

**Figure 2:**
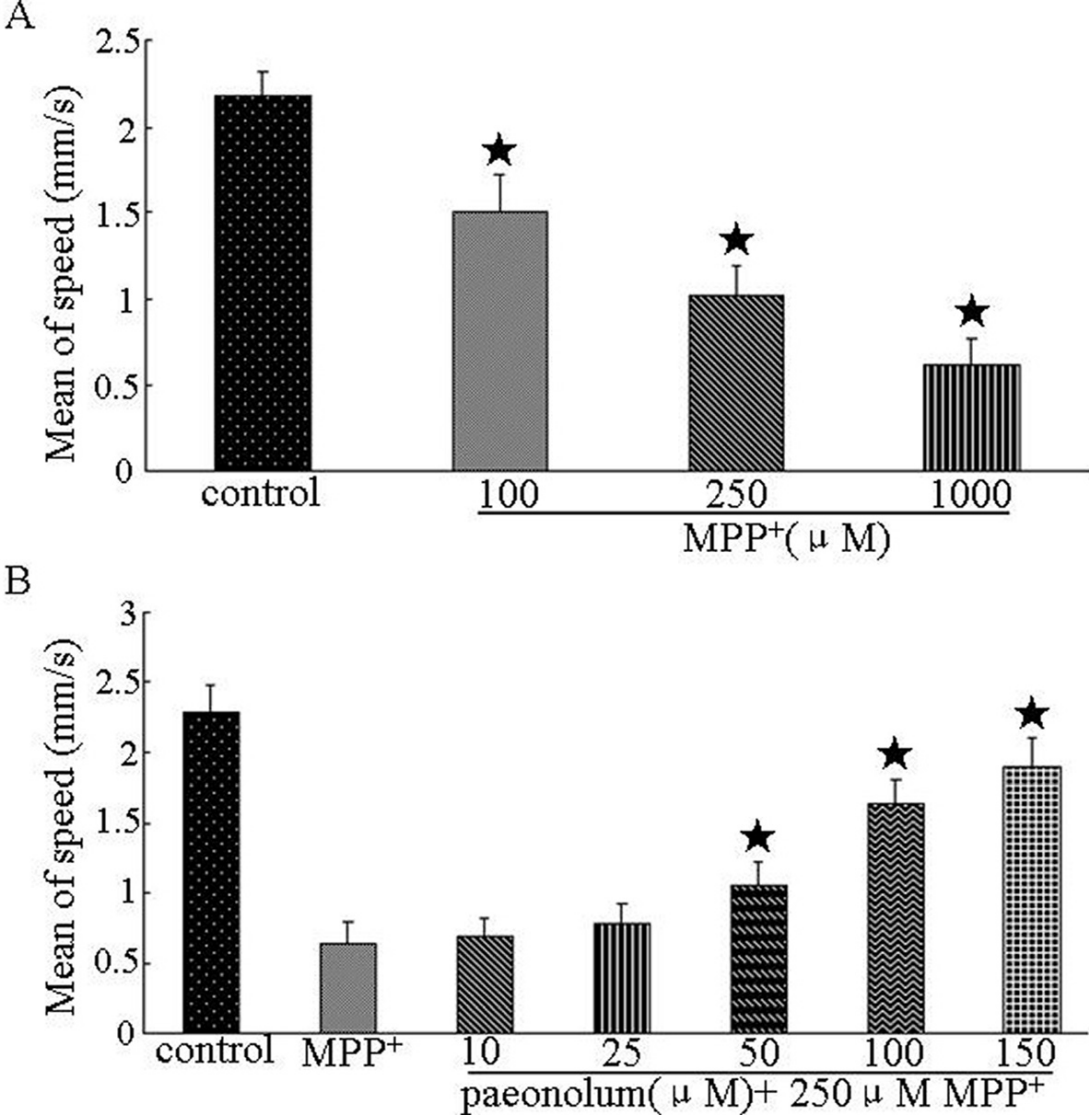
Paeonolum attenuated the MPP^+^-induced decrease in the locomotor activity of larval zebrafish. **(A)** The locomotor activity was measured as the speed of control fish and fish exposed to 100 and 1000 μM of MPP^+^ (n = 4 groups of 15 larvae per group). **(B)** Locomotor activity of control, MPP^+^ and paeonolum-treated zebrafish larvae (n = 4 × 15). Error bars represent S.E.M. *P < 0.01.

We first investigated the toxic effect of MPP^+^ in larval zebrafish, using different assessments including: overall morphology, number of DA neurons and locomotor activity. We first examined the overall morphological changes in larval zebrafish in the presence of MPP^+^. There were no significant morphological alterations observed in larval zebrafish receiving MPP^+^ up to1000 μM, compared with untreated controls, indicating that MPP^+^ has no obvious effect on normal embryonic development of zebrafish (Figure 1A). We further studied whether MPP^+^ had a specific toxic effect on DA neurons in the ETvmat2:GFP line of zebrafish. ETvmat2:GFP fish embryos were exposed to different concentrations of MPP^+^ for 4 days starting at 24 hpf. The GFP expression of 5 dpf embryos was viewed under a fluorescence microscope. The disappearance of GFP signal was used as a readout of cell death, because apoptotic GFP-positive cells are rapidly cleared by circulating cells [21]. Consistent with previous data, we found that MPP^+^ induced a significant loss of neurons from the anterior group of the posterior tubercular (PTa), paraventricular organ (Pa), anterior group of the posterior tubercular (PTa) and intermediate hypothalamus neural cluster (Hi) but not from the telencephalon and locus coeruleus in the forebrain of larval zebrafish. These results suggest that MPP^+^ selectively damaged DA neurons in zebrafish in a concentration-dependent manner (Figure 1B.). As shown in Figure 2, we also found that MPP^+^ decreased the locomotor activity of larval zebrafish. The decrease in locomotor activity correlated well with the loss of PT neurons. We then examined the effect of paeonolum against MPP^+^-induced toxicity in zebrafish. Compared with vehicle treatment, 50–150 μM paeonolum significantly prevented the MPP^+^-induced decrease in locomotor activity in a concentration- dependent manner (P < 0.01).

### Paeonolum reduced MPP^+^-induced PC12 cell damage in a concentration-dependent manner

**Figure 3:**
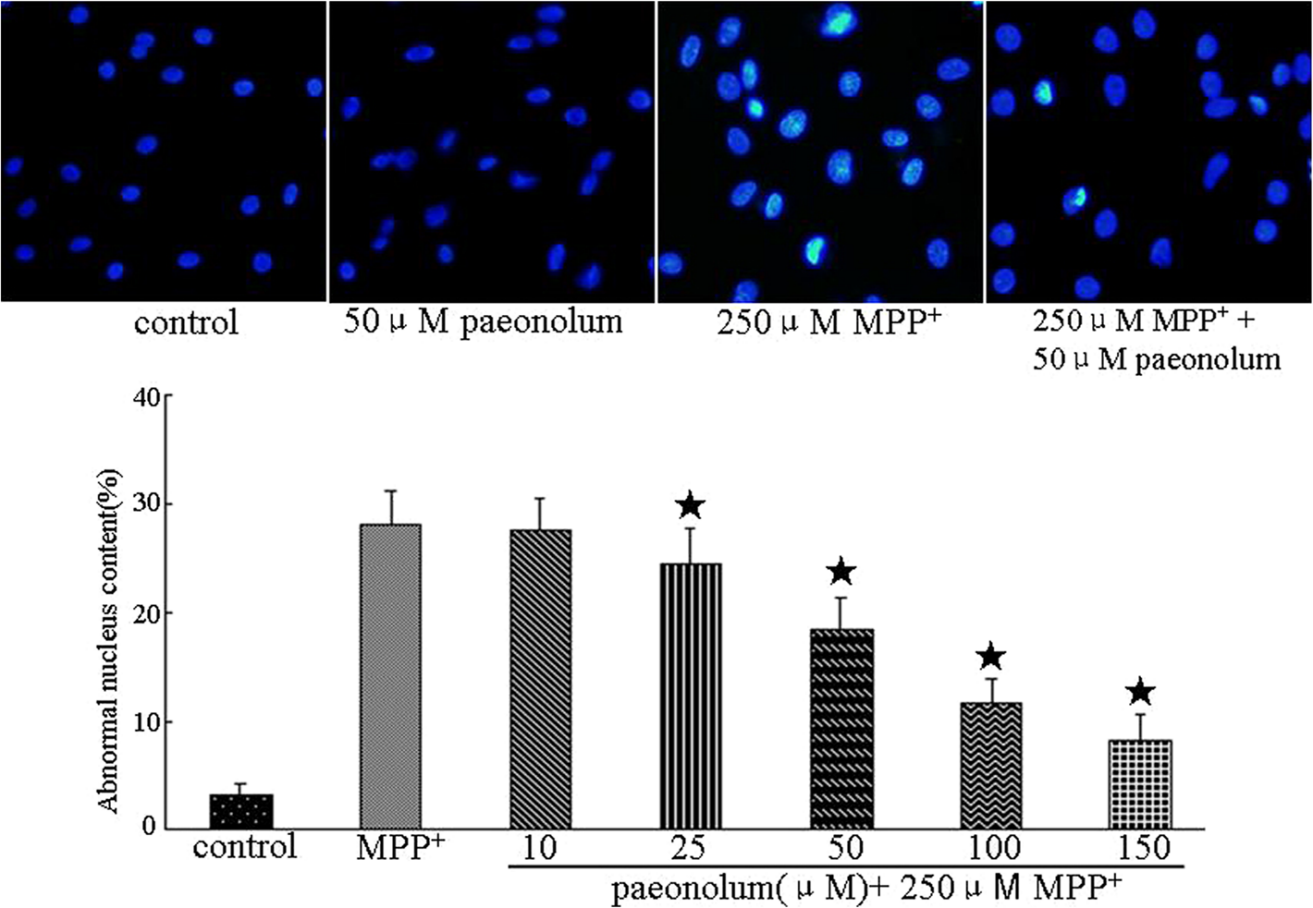
Paeonolum protected against MPP^+^-induced neurotoxicity in cultured PC12 cells. Cells were preincubated with paeonolum for 30 min and then exposed to 250 μM MPP^+^ for an additional 24 h. Pretreatment with different concentrations of paeonolum significantly attenuated MPP^+^-induced PC12 cell damage. Cell viability is indicated by MTT values of each experimental group and are expressed as a percentage of the of control group. Cellular morphological changes were detected by inverted microscopy. Representative images were selected from four independent experiments. Bar graph shows the percentage of cells with abnormal nuclei content in different experimental groups. Values are means ± SEM. *P < 0.01 compared with vehicle control. The results were obtained from four independent experiments performed in triplicate.

The viability of PC12 cells was evaluated using the MTT assay and analysis of nuclear morphology. We first examined whether paeonolum has any effect on the viability of PC12 cells under normal culture conditions. Paeonolum (10–150 μM) did not affect PC12 cell viability when compared with vehicle-treated cells (data not shown). We examined whether paeonolum could protect PC12 cells against MPP^+^ toxicity. The relative MTT values in MPP^+^-treated cells were 59.48 ± 8.12% in the vehicle group and 61.22 ± 10.25%, 64.81 ± 10.74%, 70.76 ± 9.45%, 78.21 ± 7.84% and 82.85 ± 8.71% in cells treated with paeonolum used at 10, 25, 50, 100 or 150 μM, respectively. Compared with vehicle treatment, paeonolum (50–150 μM) significantly prevented the MPP^+^-induced reduction in MTT values in a concentration-dependent manner (P < 0.01). Similar results were obtained with the morphological analysis of cell nuclei. There were substantial morphological changes such as crenation and condensation in PC12 cells exposed to MPP^+^. Pretreatment with paeonolum significantly attenuated the MPP^+^-induced morphological changes in nuclei of the PC12 cells (Figure 3).

### Paeonolum reduced MPP^+^-induced intracellular ROS

**Figure 4:**
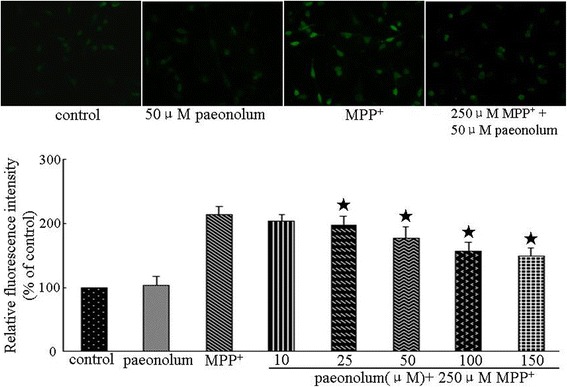
Paeonolum reduced MPP^+^-induced intracellular ROS production in PC12 cells. Cells were preincubated with paeonolum for 30 min and then exposed to 250 μM MPP^+^ for a further 24 h. Pretreatment with different concentrations of paeonolum significantly attenuated MPP^+^-induced intracellular ROS at 24 h following MPP^+^. Intracellular ROS is indicated by the DCF-DA values of each experimental group and are expressed as percentage of the control group. ROS was detected using DCF-DA. Representative images were selected from three independent experiments. Quantitative data are presented in the bar graph. Values are means ± SEM. *P < 0.01 compared with vehicle control. The results were obtained from three independent experiments performed in triplicate.

We further investigated the effect of paeonolum on MPP^+^-induced ROS production using DCF-DA, a specific dye for detecting levels of intracellular ROS. The results were expressed as percentage of fluorescence (% DCF-DA) relative to untreated controls. The relative intracellular ROS level in paeonolum alone treated cells was 102.77 ± 14.37%. Compared with control, paeonolum alone did not have any effect on basal ROS levels. The relative ROS levels in MPP^+^-treated cells were 213.12 ± 12.96% in the vehicle alone, 203.13 ± 10.37%, 196.87 ± 14.70%, 177.86 ± 16.69%, 157.37 ± 13.23% and 149.38 ± 11.98% in treatment groups with paeonolum used at 10, 25, 50, 100 and 150 μM, respectively. Compared with vehicle treatment, paeonolum (25–150 μM) significantly attenuated MPP^+^-induced ROS production in a concentration-dependent manner. The intensities of DCF-DA were dramatically increased following MPP^+^ for 24 h, whereas pretreatment with paeonolum significantly ameliorated the MPP^+^-induced increase in DCF-DA intensity (Figure 4).

### Paeonolum attenuated MPP^+^-induced reduction in mitochondrial membrane potential

To examine whether paeonolum had a protective effect in mitochondria, we detected the mitochondrial membrane potential using rhodamine 123. Treatment of PC12 cells with 250 μM MPP^+^ for 24 h produced a significant loss in mitochondrial membrane potential compared with normal controls. The relative fluorescence values of rhodamine 123 in MPP^+^-treated cells were 55.43 ± 9.63% in the vehicle alone group, 57.83 ± 11.25%, 65.23 ± 10.21%, 59.37 ± 10.36%, 65.23 ± 10.21%, 77.08 ± 10.87% and 83.18 ± 11.27% in treatment groups with paeonolum used at 10, 25, 50, 100 or 150 μM, respectively. Compared with vehicle treatment, paeonolum (50–150 μM) significantly attenuated the MPP^+^-induced reduction of mitochondrial membrane potential in a concentration-dependent manner.

### Paeonolum restored MPP^+^-induced depletion of total GSH level

**Figure 5:**
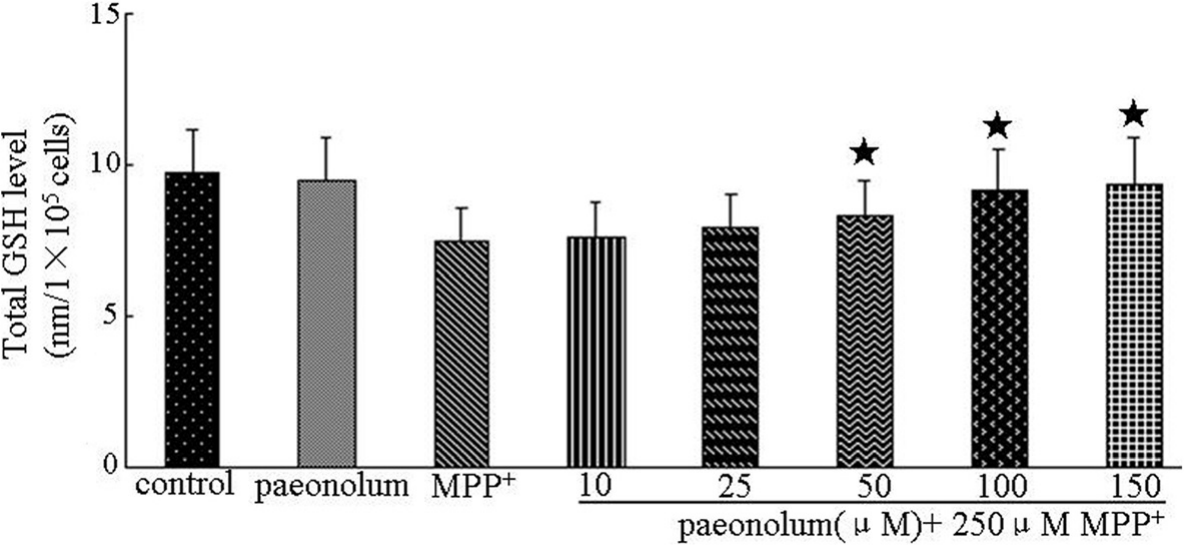
Paeonolum restored the MPP^+^-induced decrease in the total level of GSH in PC12 cells. Cells were preincubated with paeonolum for 30 min and then exposed to 250 μM MPP^+^ for further 24 h. Pretreatment with different concentrations of paeonolum significantly attenuated MPP^+^-induced decrease in total GSH level at 24 h following MPP^+^. The total level of GSH (reduced form GSH + oxidized form GSSG) was determined using glutathione reductase. Quantitative data are presented in the bar graph. Values are means ± SEM. *P < 0.01 compared with vehicle control. The results were obtained from three independent experiments performed in triplicate.

The total GSH levels were 9.74 ± 1.45 and 9.49 ± 1.44 nm/1 × 10^5^ cells, in the control and paeonolum-alone treated PC12 cells, respectively. Compared with control, paeonolum alone did not have any effect on basal GSH level. The total level of GSH was decreased to 7.45 ± 1.16 nm/1 × 10^5^ cells following incubation with 250 μM MPP^+^ for 24 h. The total GSH levels in MPP^+^-treated cells were 7.63 ± 1.11, 7.91 ± 1.17, 8.31 ± 1.16, 9.18 ± 1.36 and 9.37 ± 1.54 nm/1 × 10^5^ cells in groups treated with paeonolum at 10, 25, 50, 100 and 150 μM, respectively. Compared with vehicle treatment, paeonolum (50–150 μM) significantly restored the MPP^+^-induced depletion of total level of GSH in a concentration-dependent manner (Figure 5).

### Paeonolum against MPP^+^-induced neurotoxicity involves the intrinsic mitochondrial pathway

**Figure 6:**
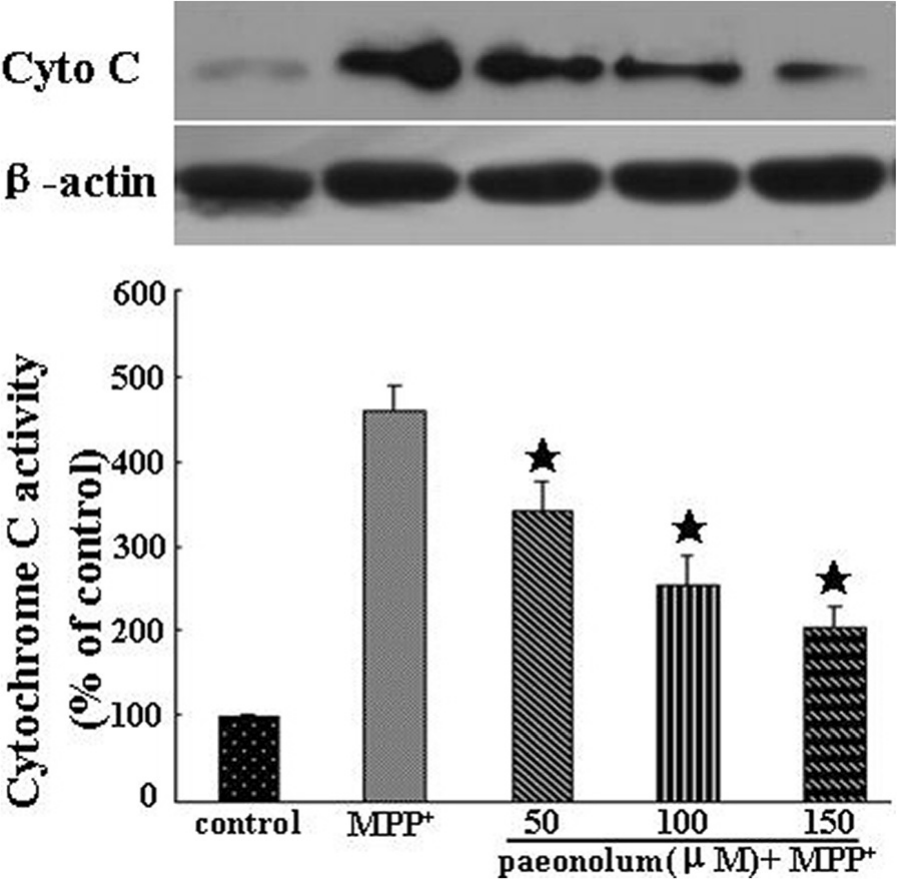
Paeonolum inhibited MPP^+^-induced the release of cytochrome c. PC12 cells were treated with 250 μM MPP^+^ for 24 h in the presence or absence of paeonolum (50, 100, 150 μM). Cytochrome c release was determined by Western blot analysis. The amount of cytochrome c was estimated by densitometric analysis of each protein band. The data are represented as means ± SEM for three independent experiments. *P < 0.01 vs. group treated with MPP^+^ alone.

**Figure 7:**
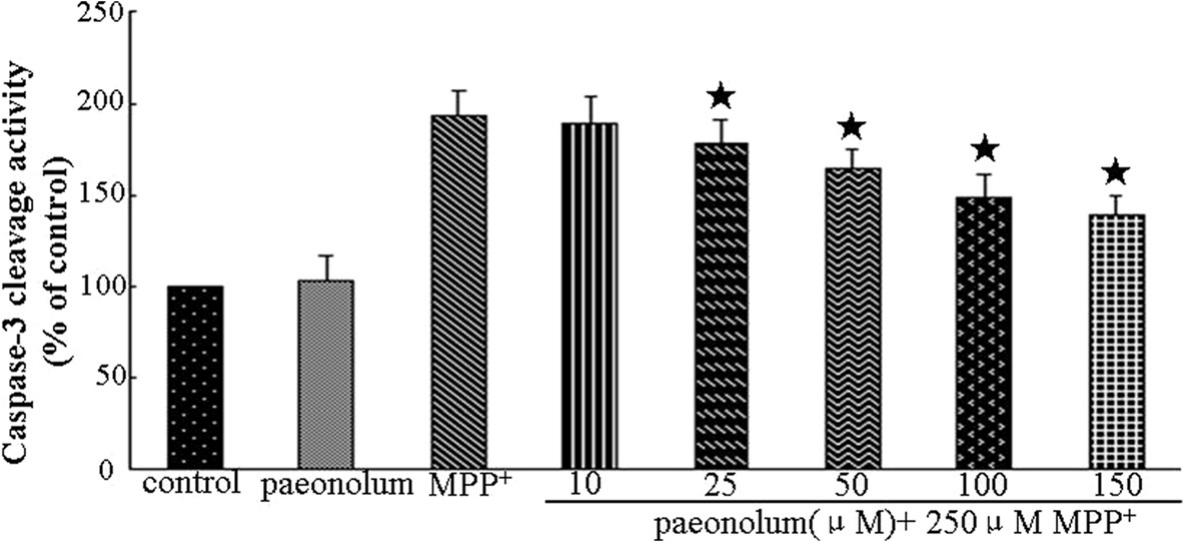
Paeonolum inhibited MPP^+^-induced increase in caspase-3 activity. PC12 cells were treated with 250 μM MPP^+^ for 24 h in the presence or absence of paeonolum (50, 100, 150 μM). The data are represented as means ± SEM for three independent experiments. *P < 0.01 vs. group treated with MPP^+^ alone.

The intrinsic mitochondrial pathway of cell death includes cytochrome c release and activation of caspase-3 and is the major death pathway involved in MPP^+^-induced toxicity. We first examined the potential effect of paeonolum on the MPP^+^-induced cytochrome c release from mitochondria using western blotting. As shown in Figure 6, MPP^+^ caused a significant (4.5-fold) increase in cytochrome c release compared with untreated controls. In contrast, paeonolum dose-dependently attenuated MPP^+^-mediated cytochrome c release. The release of cytochrome c was inhibited by 25.5%, 45.5% and 53.4% in MPP^+^ along with 50, 100 or 150 μM treatment using paeonolum, respectively. We further tested the effect of paeonolum on MPP^+^-induced activation of caspase-3 using the caspase-3 activity assay kit. As shown in Figure 7, caspase-3 activity was 193.13 ± 13.75% in the vehicle alone, 188.87 ± 15.45%, 177.87 ± 13.72%, 163.75 ± 11.12%, 148.63 ± 12.91% and 138.25 ± 11.63% in the groups treated with paeonolum at 10, 25, 50, 100 and 150 μM, respectively. Compared with vehicle treatment, MPP^+^ significantly increased caspase-3 activity compared with untreated controls. In contrast, treatment with paeonolum at 25 and 150 μM significantly attenuated MPP^+−^induced caspase-3 activity. Our results suggest that treatment with paeonolum inhibits MPP^+^-induced activation of caspase-3.

## Discussion

In the present study, we have examined the protective effects of paeonolum against MPP^+^- induced toxicity in a transgenic zebrafish line (ETvmat2:GFP) and PC12 cells. Zebrafish are an excellent model for studying PD because their nervous system shares many similarities with humans [22]. In zebrafish, PT neurons in the posterior tuberculum of the ventral diencephalon are equivalent to the nigrostriatal system in humans. In addition, the pathways involved in processing, packaging and transport of DA are also conserved between zebrafish and humans. Similar to mammals, DA neurons in zebrafish take up MPP^+^, mainly through the high-affinity DA transporter. The accumulation of MPP^+^ inside neurons in turn inactivates the mitochondrial complex I of the respiratory chain and induces cell death. In the transgenic zebrafish ETvmat2:GFP line, most monoaminergic neurons, are labeled by GFP allowing clear observation of MPP^+^-induced DA neurodegeneration in living animals. Consistent with previous observations, we found that MPP^+^ induced a large loss of PT neurons, indicating selective damage to DA neurons in a concentration-dependent manner. Furthermore, MPP^+^ also reduced locomotor activity in the zebrafish. Interestingly, the reduction of locomotor activity was closely associated with a loss of PT neurons in MPP^+^ -treated zebrafish. Given that locomotor activity in zebrafish can be easily monitored and measured on a large scale, using an automated computer tracking system, this model may be good for high throughput screening of drugs to treat PD.

To explore whether the findings in zebrafish can be translated into a future clinical approach, we investigated the mechanisms underlying the neuroprotection of paeonolum in PC12 cells. PC12 cells were used to established a MPP^+^-induced cellular model of PD because PC12 cells exhibit similar characteristics to DA neurons and have some of the physiological properties of DA neurons [23,24]. We found that incubation of PC12 cells with MPP^+^ at 250 μM for 24 h induced up to 50–60% of PC12 cell death which is in agreement with previous studies [10,25]. Similar to the findings in our zebrafish model using MPP^+^, paeonolum reduced MPP^+^-induced PC12 toxicity in a concentration-dependent manner, indicating that zebrafish can serve as a valuable model for translational research in the field of PD.

Substantial clinical and experimental evidence has demonstrated the critical role of mitochondrial dysfunction in the pathogenesis of PD [2,6]. Mitochondria are the major source of ROS production. MPP^+^ is a mitochondrial toxin and specifically inhibits mitochondrial complex I, which further promotes the opening of the mitochondrial permeability pore and causes the collapse of the mitochondrial membrane potential, causing excessive generation of ROS [1,14,26]. Excessive ROS production can damage the respiratory chain and further stimulate ROS, forming a vicious cycle resulting in cell death. Consistent with this idea, MPP^+^ significantly reduced the mitochondrial membrane potential in PC12 cells, whereas pretreatment with paeonolum attenuated the degree of MPP^+^-induced loss of mitochondrial membrane potential, suggesting that paeonolum may have a direct protective action on mitochondria. It is generally believed that the neurotoxic effects of MPP^+^ are mediated by oxidative stress. Although the antioxidant activity of paeonolum has been demonstrated in different systems, it is not clear whether antioxidant activity of paeonolum is also contributed to its neuroprotective effect in our model of MPP^+^ toxicity. We found that paeonolum attenuated MPP + −induced ROS production in a concentration-dependent manner. In addition, paeonolum also rescued the reduction of the activity of the intracellular antioxidant GSH. Given that paeonolum did not have any direct effect on GSH activity on its own, this beneficial effect may have been indirectly related to its action as a general antioxidant.

Pathologically, PD is caused by a selective loss of DA neurons in the SN. Increasing evidence from human studies on postmortem brains from PD patients, suggests that apoptotic cell death is most likely contribute to the neuronal loss observed in PD. Similarly, MPP^+^ induces apoptosis in both animals and cell cultures via affecting the mitochondrial pathway. As an inhibitor of mitochondrial complex I, MPP^+^ triggers opening of the mitochondrial permeability transition pore, induces release of apoptotic proteins such as cytochrome c into the cytoplasm and initiates caspase-3 activation, finally resulting in biochemical and morphological alterations suggestive of apoptosis. We found that paeonolum significantly suppressed MPP^+^-induced the loss of mitochondrial membrane potential, cytochrome c release and caspase-3 activity, suggesting that paeonolum is able to attenuate MPP^+^-induced toxicity through inhibition of the mitochondrial cell death cascade.

## Conclusion

Here, we have demonstrated the neuroprotective action of paeonolum against MPP^+^ toxicity in zebrafish and PC12 cells. Paeonolum exerts its protective action through reducing overproduction of ROS, attenuating loss of mitochondrial membrane potential and restoring GSH levels in MPP^+^-treated PC12 cells. The low toxicity, antioxidant properties and ease to cross the BBB warrant further investigation of this compound.
